# Publisher Correction: Engineering bacterial vortex lattice via direct laser lithography

**DOI:** 10.1038/s41467-018-07443-z

**Published:** 2018-11-19

**Authors:** Daiki Nishiguchi, Igor S. Aranson, Alexey Snezhko, Andrey Sokolov

**Affiliations:** 1 Pathogenesis of Vascular Infections Unit, Institut Pasteur, 75015 Paris, France; 2 Service de Physique de l’Etat Condensé, CEA, CNRS, Université Paris-Saclay, CEA-Saclay, 91191 Gif-sur-Yvette, France; 3 Department of Physics, The University of Tokyo, Hongo 7-3-1, Tokyo, 113-0033 Japan; 4 Department of Biomedical Engineering, Pennsylvania State University, University Park, PA 16802 USA; 5 Materials Science Division, Argonne National Laboratory, Argonne, IL 60439 USA

Correction to: *Nature Communications* **9**, 10.1038/s41467-018-06842-6; published online 26 October 2018

The original version of this Article contained errors in Fig. 2. In Fig. 2d, the label below the blue circle incorrectly read “*S*_i,a_(*t*) < 0” and should have read “*S*_i,a_(*t*) > 0”. Furthermore, the sequence of labels on the side of the bottom three panel in Fig. 2 from top to bottom incorrectly read “*S*_9,70_ > 0, *S*_9,70_ > 0, *S*_9,70_ < 0”, and should have read “*S*_9,70_ < 0, *S*_9,70_ > 0, *S*_9,70_ < 0”. Finally, in the legend to Fig. 2, the scale bar size description “Scale bar: 100 μm” was incorrectly placed in the description of panel c, and should have been placed in the description of panel d. These errors have been corrected in both the PDF and HTML versions of the Article.

