# Supplementary material for: Engineering bacterial vortex lattice via direct laser lithography
Source: Nat Commun. 2018 Oct 26;9:4486. doi: 10.1038/s41467-018-06842-6 (PMC6203773; doi:10.1038/s41467-018-06842-6)
Supplement: Supplementary file 1 — Supplementary Information [file 41467_2018_6842_MOESM1_ESM.pdf]

Supplementary Information

Engineering bacterial vortex lattice via direct laser lithography

Nishiguchi et al.

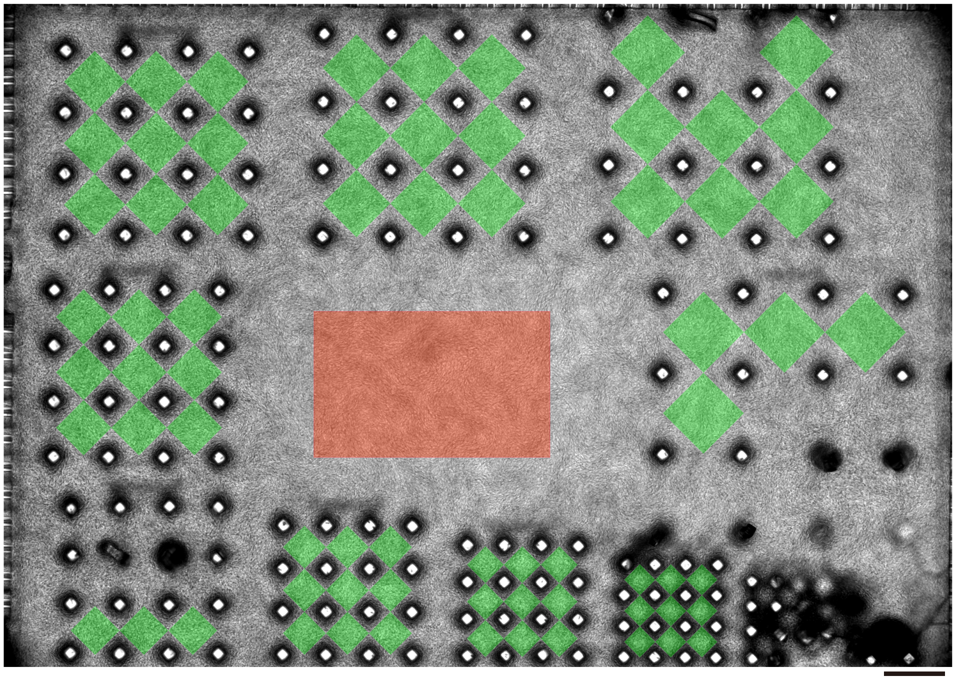

Supplementary Figure 1. The square ROIs used for analysis. Green regions for pillar lattices, and the red region for unconstrained bulk bacterial turbulence. Scale bar: 50 μm.

I. SUPPLEMENTARY NOTE 1: SQUARE LATTICE EXPERIMENT

Experimental procedure & Setup

The bacteria *Bacillus subtilis* (strain 1085) were grown in a Terrific Broth (TB) medium and concentrated by centrifugation at final concentration of 10^{10} cm^{-3}. A small drop of concentrated suspension was placed on a glass slide with the array of microscopic vertical pillars in such a way that the thickness of the drop was slightly smaller than the height of the pillars. The drop on the slide was enclosed by a plastic spacer and a coverslip with an air gap of ≈ 0.5 mm. The enclosure minimized evaporation of water still providing oxygen to bacteria. After enclosure, the whole experimental cell is inverted so that the bacteria accumulate at the surface of the suspension due to gravity and aerotaxis. The dynamics of bacteria was captured by an Olympus IX71 inverted microscope and a high-resolution (5120×3840) HS20000C camera at 10× magnification at 32.7 fps. We used only the red pixels of this RGB color camera for analysis because the images acquired by the red pixels (longer wavelength) had the highest spatial resolution due to smaller amount of scattering and diffraction of transmitted light. By enlarging the aperture stop of the microscope and decreasing the focus depth, we captured the turbulent dynamics of bacteria only at the surface. The experiments could run for minutes, but the speed of bacterial turbulence gradually decelerates. Therefore, we used the first 61 seconds of movie (2000 frames) for analysis, in which we have confirmed that the properties of unconstrained turbulence (the red region in Supplementary Figure 1) could be regarded as steady (see Supplementary Figure 5).

The pillars were 3D-printed by direct laser lithography on Photonic Professional GT system from Nanoscribe GmbH with the spatial resolution of 0.5 μm. Being 150-μm-tall and 20-μm-wide in diagonal lines, the pillars are arranged in 9 square lattices of the period a ranging from 50 μm to 130 μm with 10-μm increment (Supplementary Figure 2). The central part of the experimental cell is free of pillars. This area was used to measure parameters of unconstrained bacterial motion as a reference. To avoid a weird meniscus at the periphery of the suspension that affects the flatness of the observed surface and the behavior of outer regions, the region of printed pillars were surrounded by a wall with large tunnels through which bacteria can freely swim also 3D-printed by the laser lithography. In our experiments, we were able to track the dynamics of bacterial suspension simultaneously in all lattices with different a. That significantly reduces the noise of collected data associated with variations of bacterial swimming speed, length, fitness and age in different bacterial colonies, and makes it possible to reliably investigate how the period of structures affects the dynamics of bacterial turbulence.

The velocity field $\mathbf{v}(\mathbf{r}, t)$ of bacteria was obtained by using custom particle image velocimetry (PIV) MATLAB scripts, see Fig. 1c in the main text. The PIV subwindows were 20 μm × 20 μm and separated by every 5 μm

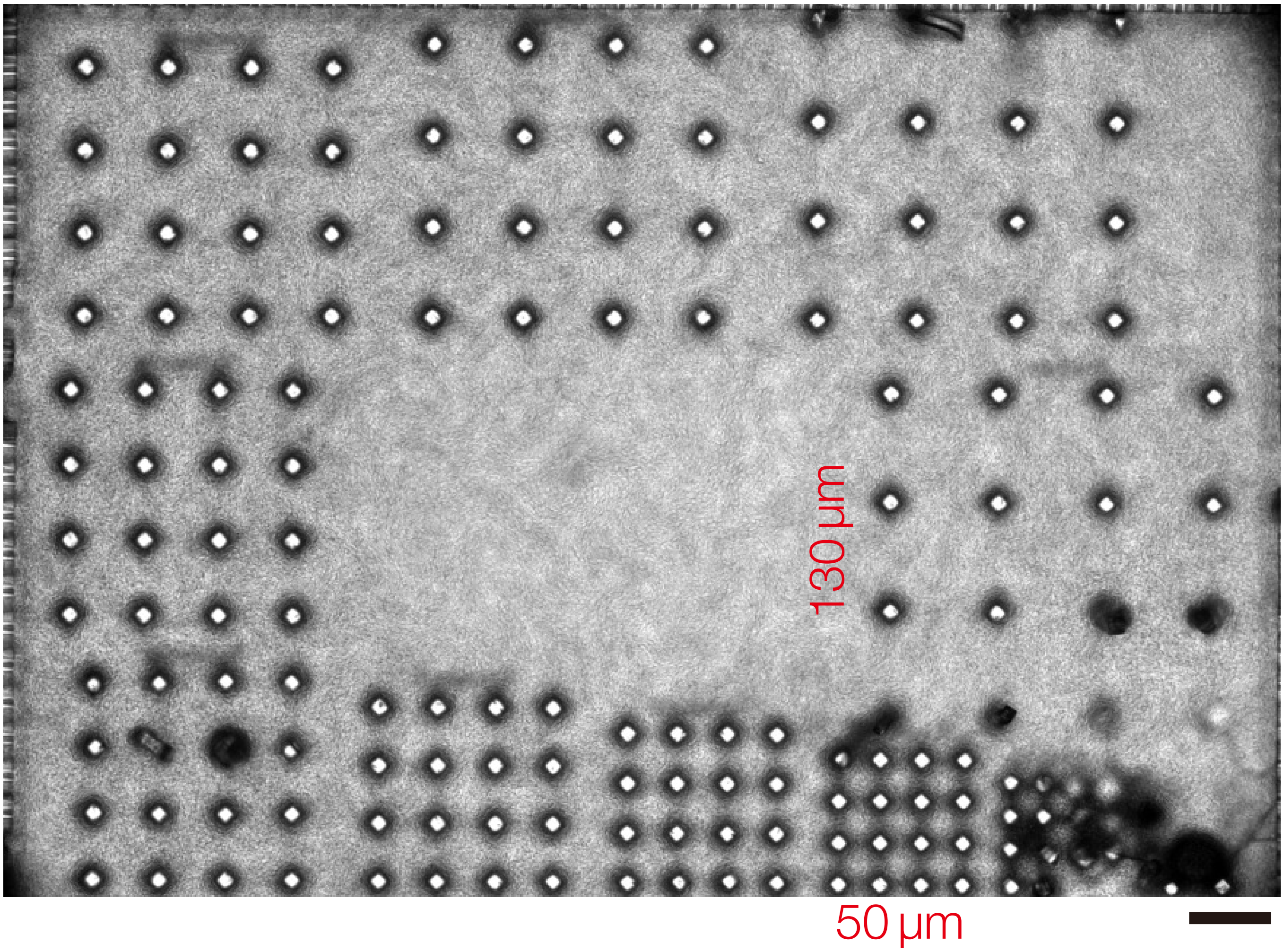

Supplementary Figure 2. Snapshot of the experiment. Sets of 4×4 pillar lattices are constructed on a glass substrate, and bacterial turbulence was introduced so that bacteria can swim between pillars. Pillar sets are organized in 9 arrays with different lattice constants a increasing from 50 μm to 130 μm (clockwise). The 40-μm lattice are excluded due to damaged pillars. The central region is left for bulk bacterial turbulence. Contrast is adjusted for visibility. Scale bar: 50 μm.

(75% overlap), the spatial resolution being smaller than any characteristic scales of the observed collective motion. Estimation of the bacterial velocity in the proximity of pillars is a technically challenging problem due to several effects: A meniscus creates an optical distortion in the vicinity of each pillars complicating bacteria tracking; the imposed geometrical confinements on bacterial motion near the pillars lead to a significant vertical motion and reduce the accuracy of spatial tracking. To avoid this problem, we excluded areas around each pillar from our analysis and measured the bacterial swimming parameters only in square regions of interest (ROI) between pillars, see Fig. 1c of the main text. We also excluded ROIs adjacent to distorted pillars, and ROIs shown in Supplementary Figure 1 are used for the following analysis.

Estimation of error bars

For quantities $F(\mathbf{r})$ such as the absolute vorticity field $|\langle \mathrm{rot}\mathbf{v}(\mathbf{r},t)\rangle_t|$ before taking spatial average $\langle\ \rangle_{\mathbf{r}\in \mathrm{ROI}_a}$, and fluctuations $\sqrt{\langle [\mathbf{v}_{\mathrm{f,t}}(\mathbf{r},t) - \langle \mathbf{v}_{\mathrm{f,t}}(\mathbf{r},t)\rangle_t]^2\rangle_t}$, error bars are estimated via the standard deviations among the ROIs with the same lattice constant a. Therefore, corresponding error bars are calculated as,

$$\sqrt{\frac{\sum_i \left[\langle F(\mathbf{r})\rangle_{\mathbf{r}\in \mathrm{ROI}_a^i} - \langle F(\mathbf{r})\rangle_{\mathbf{r}\in \mathrm{ROI}_a}\right]^2}{N(a)-1}}, \tag{1}$$

where ROI_a^i denotes the i-th ROI in the lattice with the period a, and $N(a)$ is the number of the analyzed ROIs in

the lattice with the period a.

To be more specific, error bars of the mean vorticity,

$$\langle |\langle \mathrm{rot}\mathbf{v}(\mathbf{r},t)\rangle_t|\rangle_{\mathbf{r}\in\mathrm{ROI}_a}, \tag{2}$$

are estimated as,

$$\sqrt{\frac{\sum_i \left[\langle |\langle \mathrm{rot}\mathbf{v}(\mathbf{r},t)\rangle_t|\rangle_{\mathbf{r}\in\mathrm{ROI}_a^i} - \langle |\langle \mathrm{rot}\mathbf{v}(\mathbf{r},t)\rangle_t|\rangle_{\mathbf{r}\in\mathrm{ROI}_a}\right]^2}{N(a)-1}}. \tag{3}$$

Similarly, error bars for the mean enstrophy,

$$\langle\langle [\mathrm{rot}\mathbf{v}(\mathbf{r},t)]^2\rangle_t\rangle_{\mathbf{r}\in\mathrm{ROI}_a}, \tag{4}$$

are estimated as,

$$\sqrt{\frac{\sum_i \left[\langle\langle [\mathrm{rot}\mathbf{v}(\mathbf{r},t)]^2\rangle_t\rangle_{\mathbf{r}\in\mathrm{ROI}_a^i} - \langle\langle [\mathrm{rot}\mathbf{v}(\mathbf{r},t)]^2\rangle_t\rangle_{\mathbf{r}\in\mathrm{ROI}_a}\right]^2}{N(a)-1}}. \tag{5}$$

Error bars for the velocity fluctuations of the bacterial full velocity field $\mathbf{v}_\mathrm{f}$ or the tangential velocity field $\mathbf{v}_\mathrm{t}$,

$$\sigma_{\mathrm{f,t}}(a) = \left\langle \sqrt{\langle [\mathbf{v}_{\mathrm{f,t}}(\mathbf{r},t) - \langle \mathbf{v}_{\mathrm{f,t}}(\mathbf{r},t)\rangle_t]^2\rangle_t} \right\rangle_{\mathbf{r}\in\mathrm{ROI}_a}, \tag{6}$$

are also estimated as,

$$\sqrt{\frac{\sum_i \left[\langle \sqrt{\langle [\mathbf{v}_{\mathrm{f,t}}(\mathbf{r},t) - \langle \mathbf{v}_{\mathrm{f,t}}(\mathbf{r},t)\rangle_t]^2\rangle_t}\rangle_{\mathbf{r}\in\mathrm{ROI}_a^i} - \langle \sqrt{\langle [\mathbf{v}_{\mathrm{f,t}}(\mathbf{r},t) - \langle \mathbf{v}_{\mathrm{f,t}}(\mathbf{r},t)\rangle_t]^2\rangle_t}\rangle_{\mathbf{r}\in\mathrm{ROI}_a}\right]^2}{N(a)-1}}. \tag{7}$$

Persistence of vortices: Life times

To evaluate the stability of bacterial vortices, the probability $P_a(t)$ of a spin in a lattice with the period a to remain oriented in the same direction for a period of time t is calculated. Existence of stable lattices of interacting spins complicates the spin switching dynamics and requires careful analysis. Because the vortex lattices for a = 60–90 μm is stable, there are favorable and unfavorable directions of rotation for each ROI in these lattices. We do observe that temporal fluctuations lead to rotations with unfavorable directions, but such rotations are short-lived compared with ones with favorable directions. Therefore, there exist two distinct life times τ_a^{long} and τ_a^{short} corresponding to favorable and unfavorable directions respectively. In other words, due to the hydrodynamic interaction between vortices, the short time τ_a^{short} corresponds to switching from local ferromagnetic (unstable) order to antiferromagnetic (stable) order (Supplementary Figure 3), which leads to fluctuations in the order parameter $\chi_a(t)$ (Fig. 2e in the main text). In a stable antiferromagnetic configuration, a vortex remains its orientation for a much longer period of time τ_a^{long}.

Because vortices at the edges of the lattices are affected by surrounding chaotic bacterial turbulence, we fabricated larger arrays of pillars (9×9 instead of 4×4) for periods a = 50–90 μm, see Supplementary Figure 4. To eliminate the boundary effects for obtaining the probability $P_a(t)$ accurately, we excluded the outer two layers of the ROIs from the analysis. We calculated the probability $P_a(t)$ of favorable and unfavorable directions for each ROI separately (Fig. 3b in the main text), and fitted them with $P_a(t) \propto \exp(-t/\tau_a)$. The fitting range was [0 s, 1 s] for unfavorable directions and [0 s, 15 s] for favorable directions. As a result, we obtained the life time τ_a as a function of the period a (Fig. 3c in the main text).

Properties of the pillar-free reference area

Here we summarize experimentally obtained properties of unconstrained bacterial turbulence in our reference area at the central region of the field of view without pillars, the red rectangle region in Supplementary Figure 1.

We calculated the root mean square velocity v_{rms}, the mean vorticity, and the mean enstrophy defined as follows:

$$v_\mathrm{f}^{\mathrm{rms}}(a) = \left\langle \sqrt{\langle [\mathbf{v}_\mathrm{f}(\mathbf{r},t)]^2\rangle_t} \right\rangle_{\mathbf{r}\in\mathrm{ROI}_{\mathrm{ref}}}, \tag{8}$$

$$\text{mean vorticity} = \langle\langle \mathrm{rot}\mathbf{v}(\mathbf{r},t)\rangle_t\rangle_{\mathbf{r}\in\mathrm{ROI}_{\mathrm{ref}}}, \tag{9}$$

$$\text{mean enstrophy} = \langle\langle [\mathrm{rot}\mathbf{v}(\mathbf{r},t)]^2\rangle_t\rangle_{\mathbf{r}\in\mathrm{ROI}_{\mathrm{ref}}}, \tag{10}$$

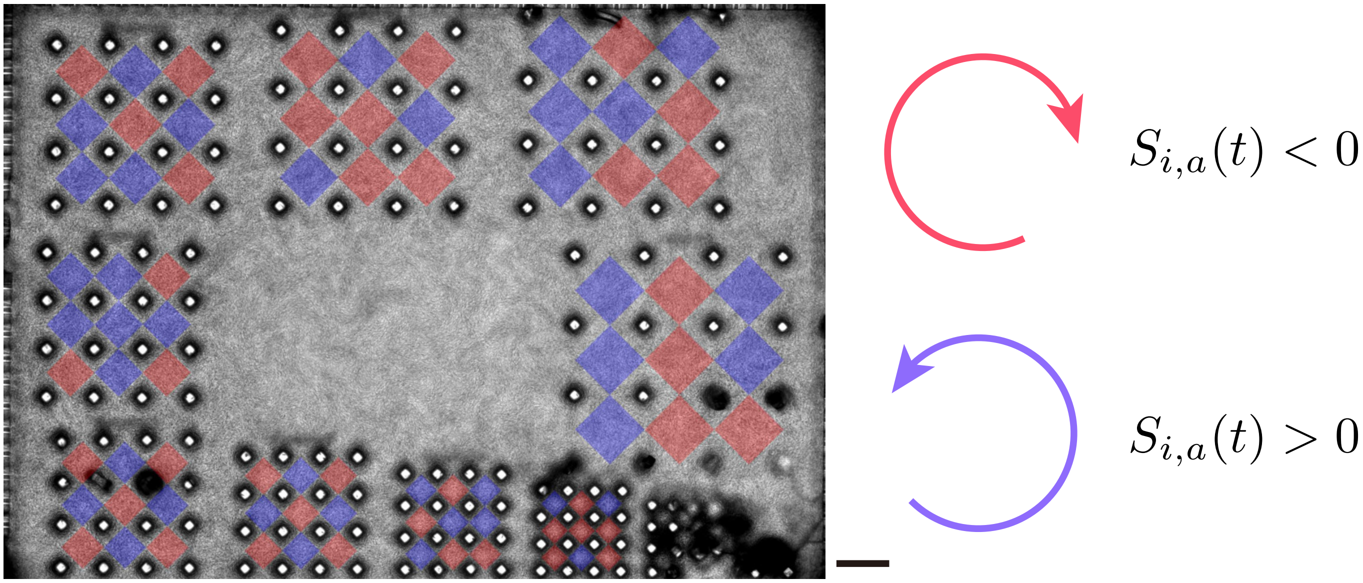

Supplementary Figure 3. Instantaneous signs of spins are overlaid on experimental snapshots. Clockwise rotations ($S_{i,a}(t) < 0$) and counterclockwise rotations ($S_{i,a}(t) > 0$) are represented by red and blue respectively. Scale bars: 100 μm.

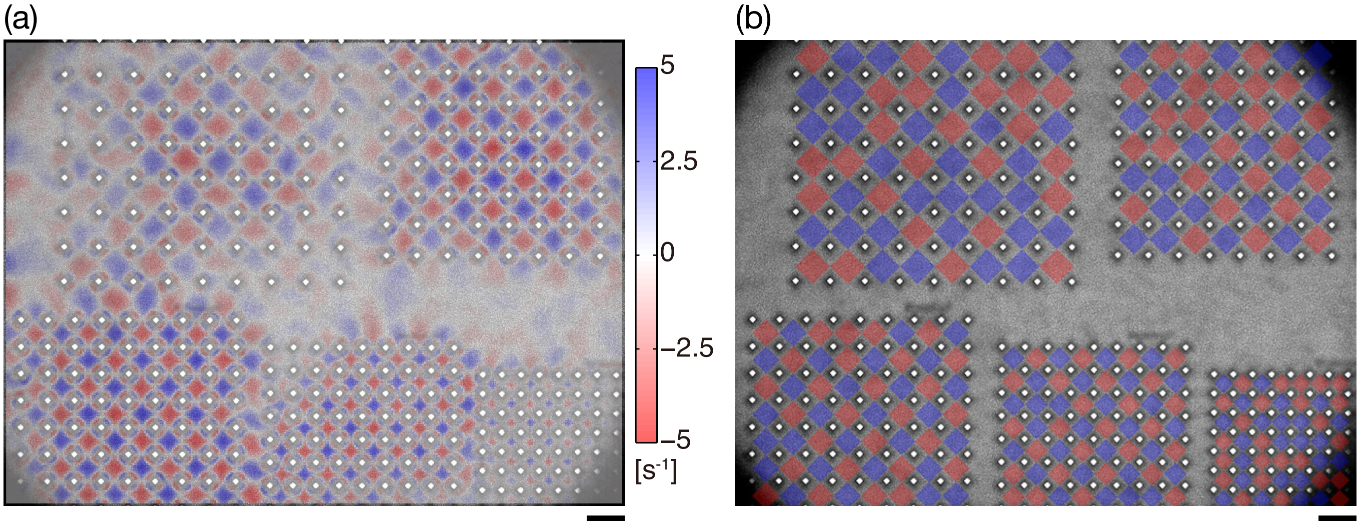

Supplementary Figure 4. Experiments on larger 9 × 9 pillar arrays. Some pillars are out of the field of view. (a) Average vorticity of bacterial velocity field. a = 50, 60, 70, 80, 90 μm. (b) Instantaneous map of spin signs. For a = 70 μm defects observed mostly at the perimeter of the lattice. Scale bars: 100 μm.

where $\mathrm{ROI_{ref}}$ represents the ROI for the bulk unconstrained turbulence, which is shown as the red rectangle in Supplementary Figure 1. We also calculated the correlation function,

$$C_\infty(r) := \frac{\langle\langle \mathbf{v}(\mathbf{r}',t)\cdot\mathbf{v}(\mathbf{r}'+\mathbf{r},t)\rangle_{\mathbf{r}',\mathbf{r}'+\mathbf{r}\in \mathrm{ROI_{ref}}}\rangle_t}{\langle\langle |\mathbf{v}(\mathbf{r}',t)|^2\rangle_{\mathbf{r}'\in \mathrm{ROI_{ref}}}\rangle_t}. \tag{11}$$

All the results are shown in Supplementary Figure 5.

Although the mean enstrophy gradually changes about $\sim 10\%$ (Supplementary Figure 5c), v_{rms} stays almost constant, which assures that our experiment was done in a steady state (Supplementary Figure 5a). The mean vorticity shown in Supplementary Figure 5b naturally stays around 0, which means there is no *a priori* favored direction of rotations. This assures that emergence of each vortex formed in the lattices is a consequence of spontaneous macroscopic chiral symmetry breaking.

From the correlation function $C_\infty(r)$, we can extract the correlation length of the unconstrained bacterial turbulence $L_\infty \simeq 45$ μm (Supplementary Figure 5d). This value was compared with the correlation lengths in the lattice structures in Fig. 3d in the main text.

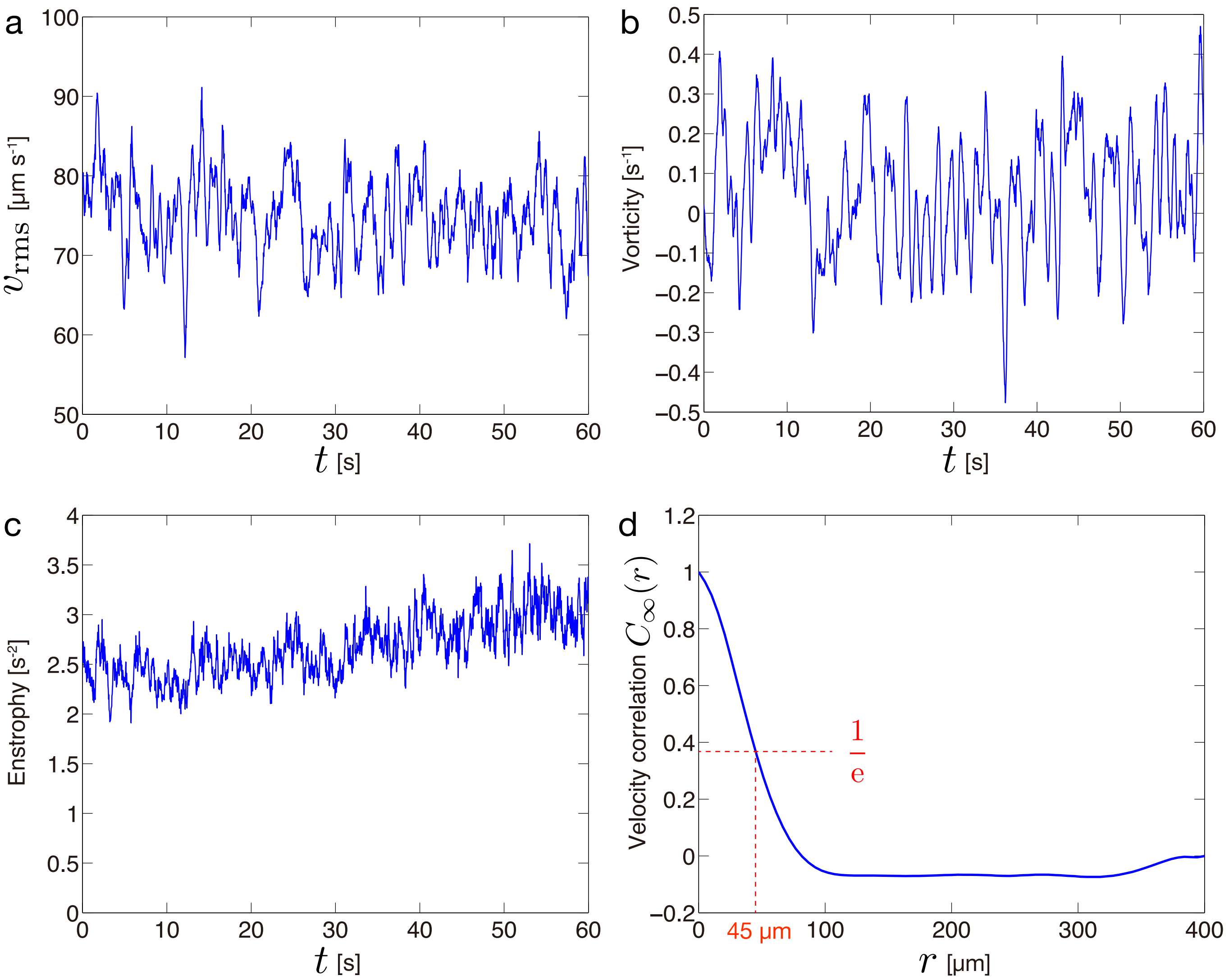

Supplementary Figure 5. Experimentally obtained properties of bulk unconstrained bacterial turbulence in the reference area $\mathrm{ROI_{ref}}$ in the red rectangle shown in Supplementary Figure 1. (a) Time series of the root mean square velocity $v_{\rm rms}$. $v_{\rm rms}$ is steady and we do not observe any discernible change. (b) Time series of the mean vorticity $\langle\langle \mathrm{rot}\mathbf{v}(\mathbf{r},t)\rangle_t\rangle_{\mathbf{r}\in\mathrm{ROI_{ref}}}$. (c) Time series of the mean enstrophy $\langle\langle [\mathrm{rot}\mathbf{v}(\mathbf{r},t)]^2\rangle_t\rangle_{\mathbf{r}\in\mathrm{ROI_{ref}}}$. (d) Velocity correlation function $C_\infty(r)$. Red dashed line represents the correlation length $L_\infty \simeq 45$ μm at which $C_\infty(r)$ becomes smaller than 1/e.

Visualization of vorticity

To gain insight into the bacterial dynamics, we visualized vorticity field of bacterial velocity field as in Fig. 1ab in the main text. Here we show instantaneous vorticity field in Supplementary Figure 6b. For this purpose, we chose the range of the color plot as $[-5\ \mathrm{s}^{-1}, +5\ \mathrm{s}^{-1}]$ so that only 1.38% of area in the field of view is saturated (Supplementary Figure 6a).

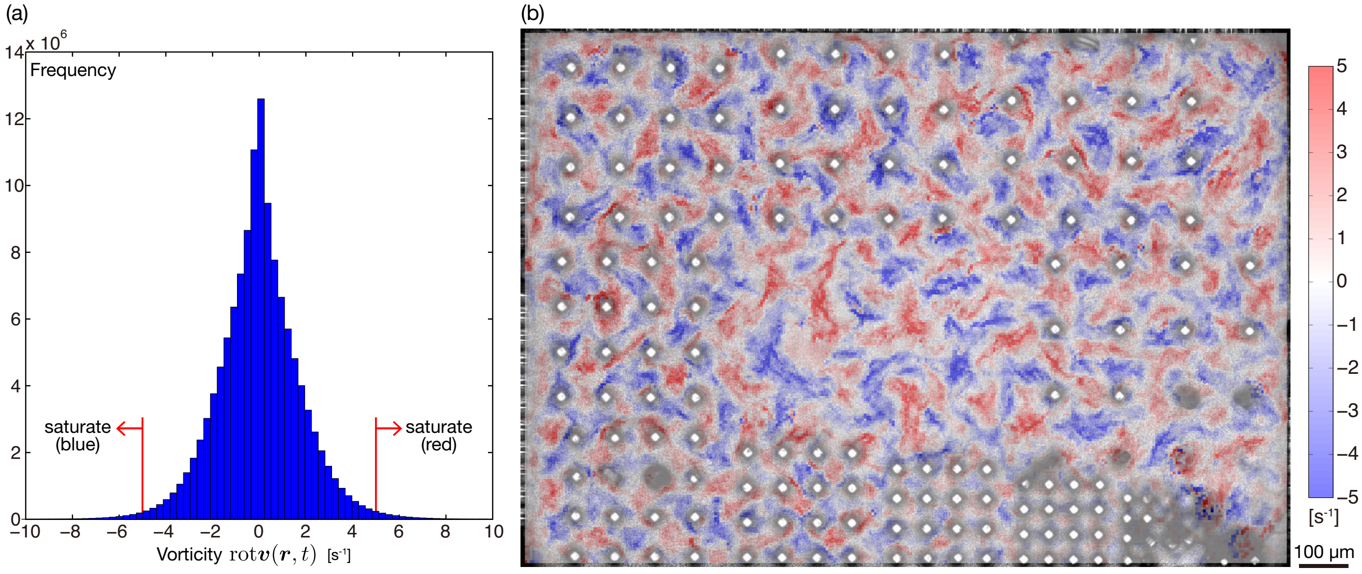

Supplementary Figure 6. (a) Histogram of vorticity calculated in all the field of view and in all the analyzed 2000 frames. We chose the threshold for color map so that only 1.38% of area is saturated. (b) Instantaneous vorticity field is overlaid on an experimental snapshot. Scale bar: 100 μm

II. SUPPLEMENTARY NOTE 2: HEXAGONAL LATTICE EXPERIMENT

While the main focus of our work was on dynamics of swimming bacteria in the square lattices of pillars, we also performed additional experiments for hexagonal lattices (Supplementary Figure 7). As we can infer from the results on square lattice experiments, hydrodynamic continuity cannot be fulfilled without imposing any frustration between spins in hexagonal lattices. In accordance with the lattice geometry, we printed hexagonal pillars for this experiment instead of square pillars used in the square lattice experiments.

Because we have obtained the strong antiferromagnetic order in the square lattices with the lattice constant $a \simeq 70$ μm, we investigated hexagonal lattices whose diagonal distance is comparable to 70 μm. In these hexagonal lattices, we defined the lattice constant a as the distance between two nearest pillars, and we chose appropriate lattice constants a so that the diameter of inscribed circles of hexagons $\sqrt{3}a \simeq 70$ μm, or $a \simeq 40.4$ μm. Therefore, we tested two lattice constants: $a = 40$ μm and $a = 45$ μm. In this experiment, we captured a movie at 52.7 fps and analyzed 4112 frames (78 seconds).

In spite of such choice of length scale that should be favorable to vortex formation, we could not observe neither ferromagnetic nor antiferromagnetic order in the hexagonal lattices (Supplementary Figure 8). We analyzed the adjacent spin correlations $\chi_a(t)$ defined in eq. (2) of the main text by defining the ROIs as shown in Supplementary Figure 9a. The spin correlations $\chi_a(t)$ fluctuate a lot for both $a = 40$ μm and $a = 45$ μm, but they almost always stay around 0 and $|\chi_a(t)| < 0.5$, which means that there is no stable emergent order. Note that $0 \ll \chi_a(t) \leq 1$ and $-1 \leq \chi_a(t) \ll 0$ correspond to ferromagnetic and antiferromagnetic order respectively.

This again seemingly contradicts with the results in Ref. [1], in which they observed strong ferromagnetic order of bacterial turbulence confined in a hexagonal lattice of a microfluidic device, and highlight the importance of slight difference in boundary conditions for understanding macroscopic behavior, especially the emergent order, of bacterial turbulence. These results demonstrate again that the vortex lattice formation is triggered in accordance with hydrodynamic continuity conditions, and the underlying mechanism of vortex lattice formation is distinctively different from the previous study [1].

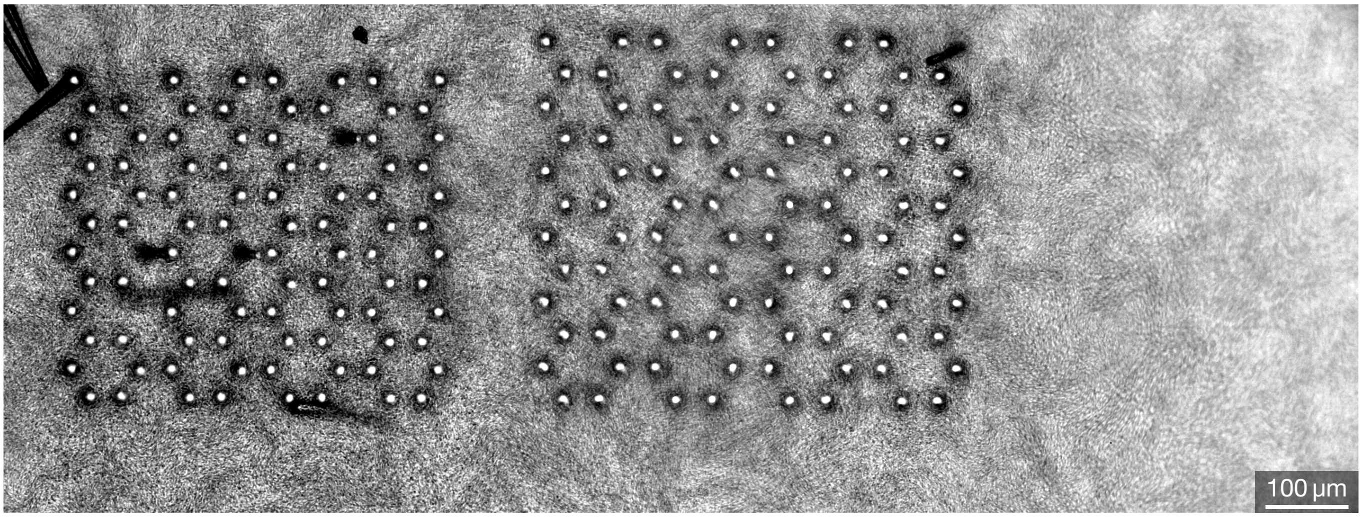

Supplementary Figure 7. Snapshot of the experiments on hexagonal lattice. The distances between the two nearest pillars, or the lattice constant, a are $a = 40$ μm for the left lattice and $a = 45$ μm for the right lattice.

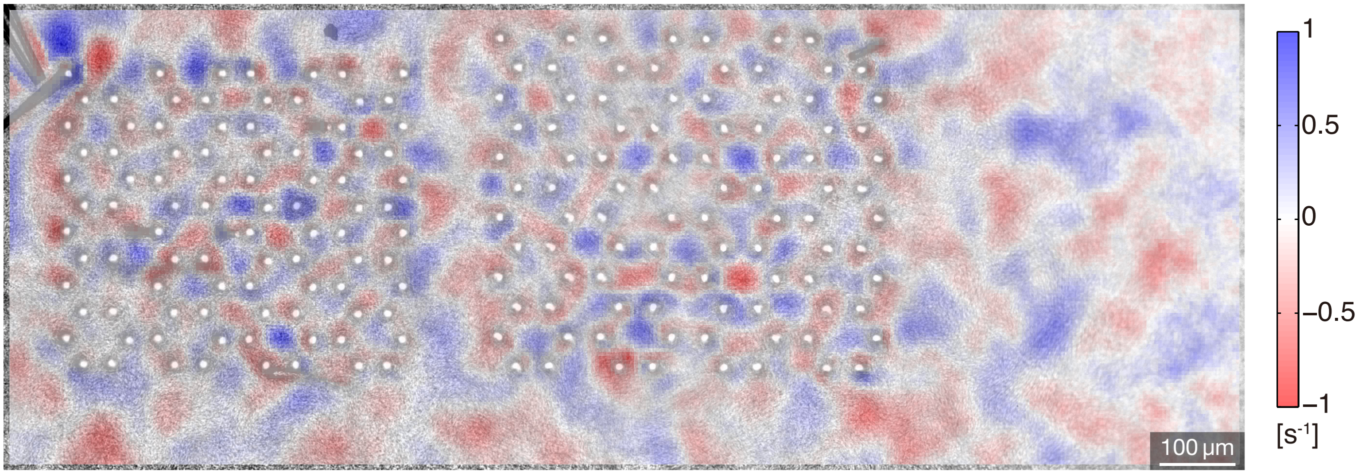

Supplementary Figure 8. Color plot of the time averaged vorticity field overlaid on the snapshot of Supplementary Figure 7. Neither ferromagnetic nor antiferromagnetic order is observed as expected.

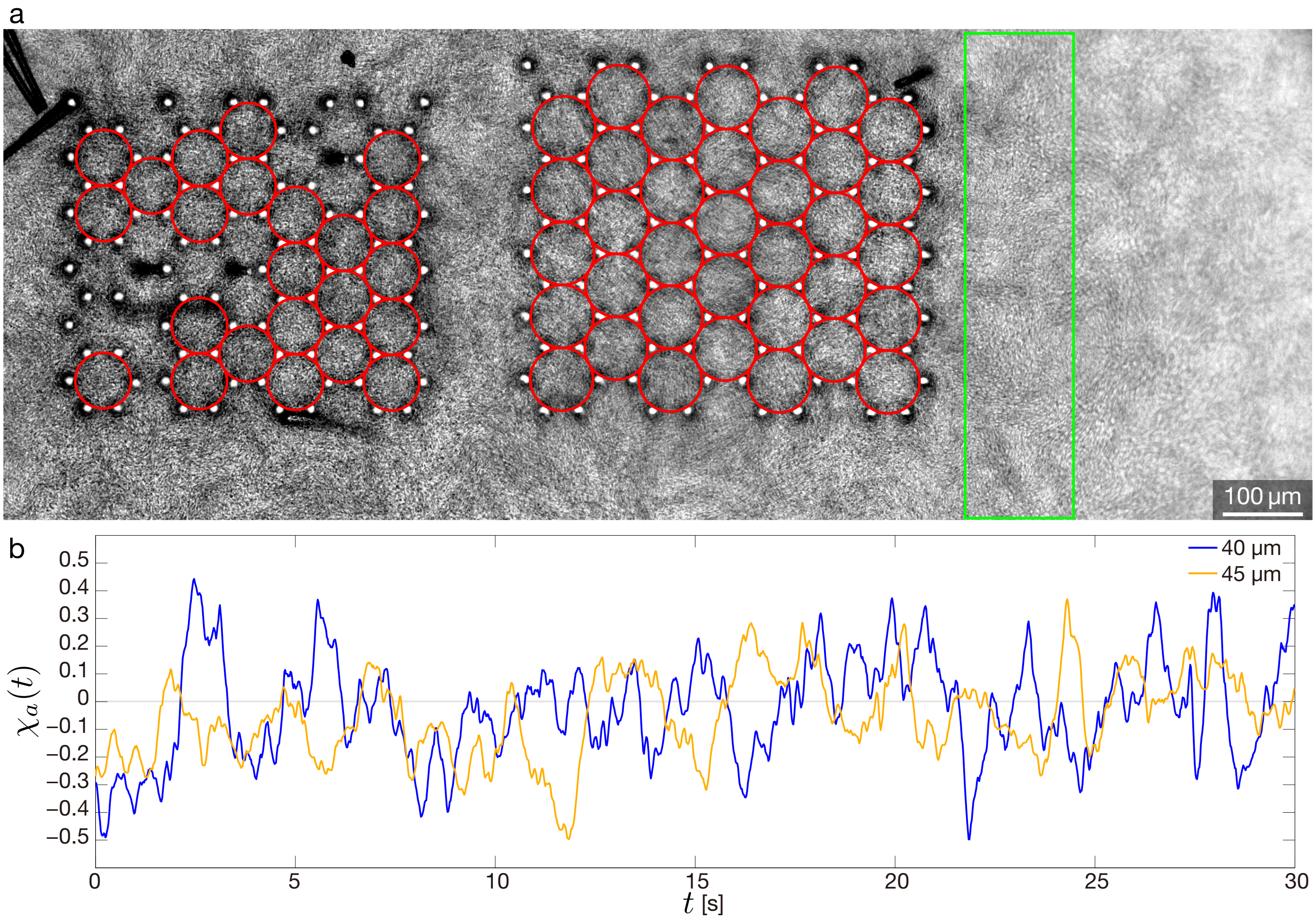

Supplementary Figure 9. (a) ROIs used for analysis. Red circular ROIs were used inside the lattices. Regions adjacent to distorted pillars were neglected for analysis. Green rectangular area represents the reference area for the unconstrained turbulence. (b) Temporal dynamics of spins $\chi_a(t)$ for different lattice constants a. Blue: $a = 40\ \mu$m. Orange: $a = 45\ \mu$m. The spin correlations $\chi_a(t)$ fluctuate around 0 by frequently changing their signs, and no clear order emerges.

III. SUPPLEMENTARY NOTE 3: LATTICE SIZE SCALING EXPERIMENT

Experimental setup

To assess how the stability of the antiferromagnetic vortex lattices depend on the size of the lattices or the proportion of the vortices on the lattice perimeters, we conducted experiments on pillar arrays with different sizes. We 3D-printed pillar arrays with the most stabilizing period $a = 70$ μm so that we can capture simultaneously the self-organized vortex lattices with their sizes $n \times n = 1 \times 1, 2 \times 2, \ldots, 7 \times 7$ as shown in Supplementary Figure 10. (Note that here n denotes the linear dimension of *vortex* lattices and that the sizes of the corresponding *pillar* arrays are $(n+1) \times (n+1) = 2 \times 2, 3 \times 3, \ldots, 8 \times 8$.) To reduce the interactions between vortex lattices in different pillar arrays, the pillar arrays were separated from each other by the distance sufficiently larger than the correlation length of the bulk bacterial turbulence ($L_\infty \simeq 45$ μm)). Due to the limited field of view of the camera (1640 μm $\times$ 1230 μm), we captured two experimental movies at different positions with overlapping fields of view. We first captured the region shown in Supplementary Figure 10b (Movie I) and then the other region shown in Supplementary Figure 10a (Movie II). We used the same microscope and the same camera as the other previous experiments, and captured the movies at 30.0 fps for 133 seconds (4000 frames).

Experimental results

First, we calculated the average vorticity field and defined the favorable direction of rotation for each spin (Supplementary Figure 11ab). Then instantaneous signs of spins in all the lattices are calculated (Supplementary Figure 11cd) and used to evaluate the persistence times of the spins τ_k for their favorable directions in the same manner as described above. Here, k denotes the numbers of neighboring spins. We calculated τ_k for the different lattice sizes (Supplementary Figure 12a), and then all the data for the same k were averaged (Fig. 4a of the main text). Error bars in Fig. 4a of the main text are standard errors calculated from the standard deviations among τ_k obtained from the different lattice sizes.

Then we calculated order parameter $\langle \chi_a \rangle_t$ for each lattice in both Movie I and Movie II. The order parameters $\langle \chi_a \rangle_t$ obtained from Movie I and Movie II of the lattice size scaling experiment (Supplementary Figure 10) and the large lattice experiment (Fig. 3 in the main text and Supplementary Figure 4) are plotted together in Supplementary Figure 12b. Figure 4c in the main text is made by taking the average of these data, and the error bars there are calculated from the error propagation law using the errors in Supplementary Figure 12b.

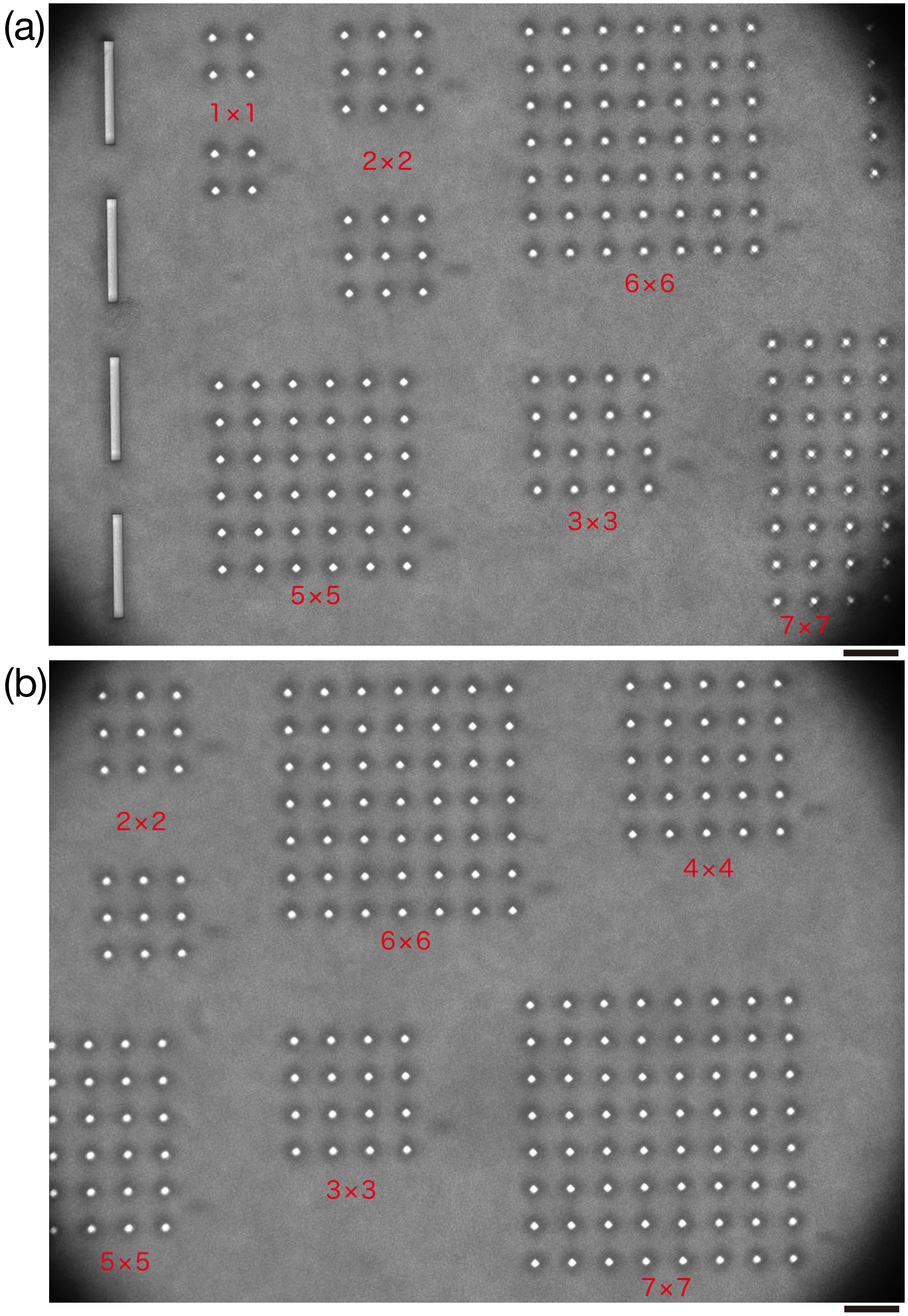

Supplementary Figure 10. Snapshots for lattice size scaling experiments. We captured two movies with overlapping fields of view successively: first Movie I for (b) and then Movie II for (a). As seen on the left side of (a), we 3D-printed walls surrounding the pillar arrays to avoid the effect of meniscus. Scale bars: 100 μm.

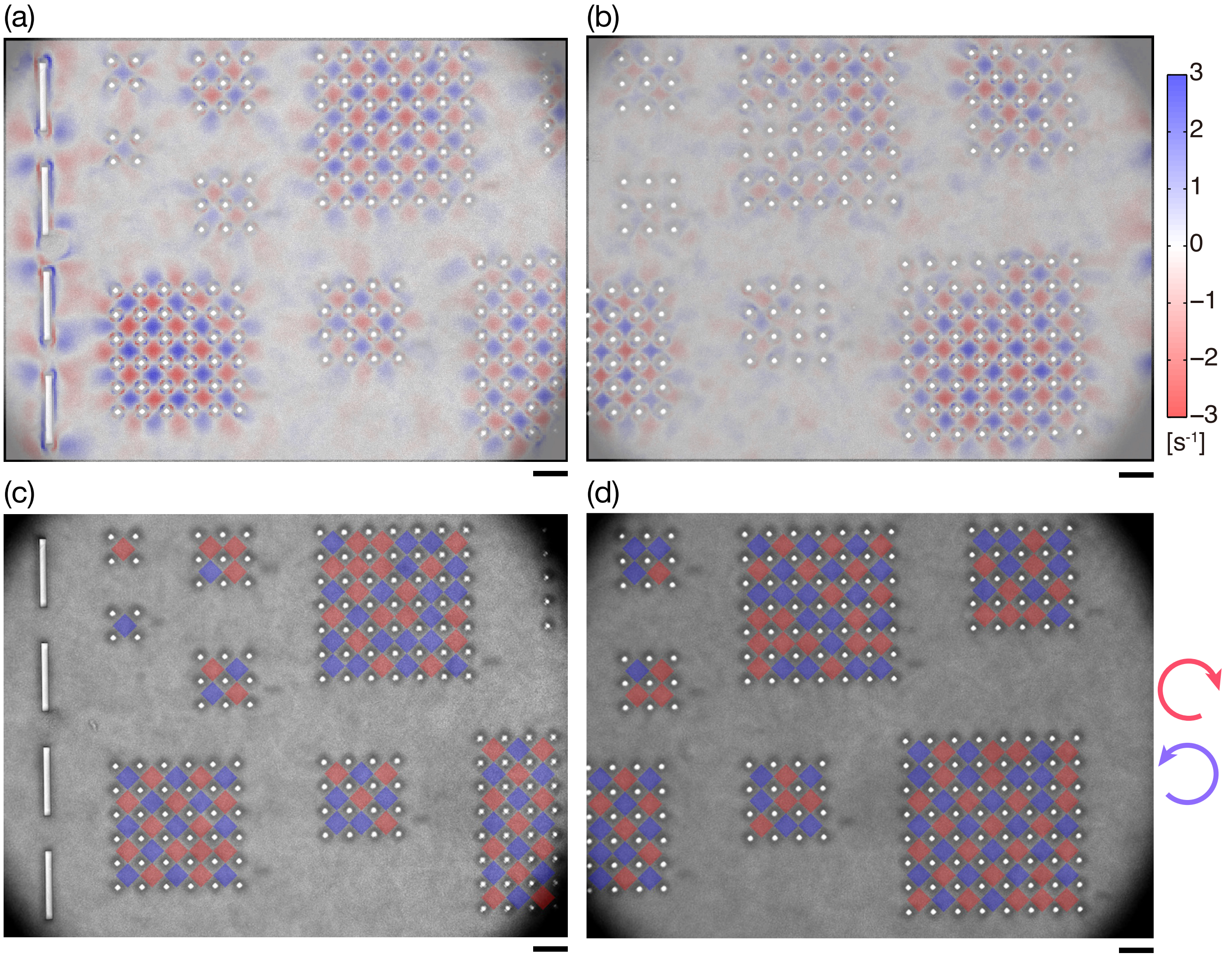

Supplementary Figure 11. (a)(b) Temporally averaged vorticity of bacterial velocity field. Larger pillar arrays exhibit stronger antiferromagnetic vortex lattice formation. Color bar is common to (a) and (b). (c)(d) Instantaneous signs of spins are overlaid on experimental snapshots in the same manner as in Supplementary Figure 3. Scale bars: 100 μm.

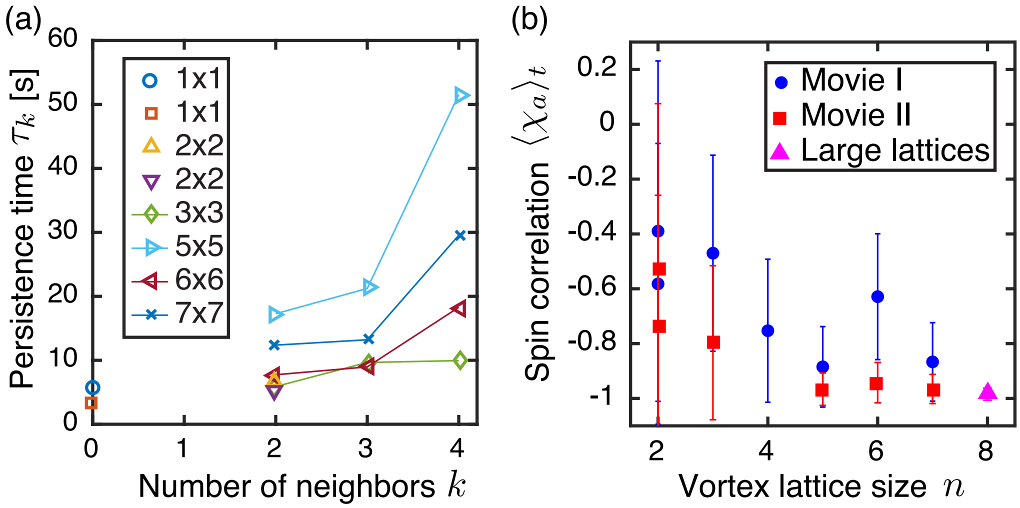

Supplementary Figure 12. (a) Persistence time τ_k of the favorable direction vs the number of neighbors k for the lattices with different sizes $n \times n$ calculated from Movie II. Average of these data is shown in Fig. 4a in the main text. (b) Order parameter (spin correlation) $\langle\chi_a\rangle_t$ vs the linear size n of the vortex lattice at the lattice constant $a = 70$ μm. Data obtained from the lattice size scaling experiment (Movies I and II, blue circles and red squares respectively) and the large 9×9 pillar array (8×8 vortices) experiments (magenta triangles) are shown together. Average of these data is shown in Fig. 4c in the main text. Error bars: standard deviations of time series of $\chi_a(t)$.

IV. SUPPLEMENTARY NOTE 4: THEORETICAL DESCRIPTION ON PERSISTENCE TIME

Our theoretical model presented in Methods section predicts exponential dependence of the persistence time τ_k on the number of neighbors k (Eq. (9) in the main text),

$$\tau_k \sim \tau_0 \exp(2\eta k/D). \tag{12}$$

This matches well with the experimental data obtained from the lattice size scaling experiment for the lattice period $a = 70$ μm. To further explore the implication of our model, we calculated the persistence time τ_k for different lattice periods a by using the data from the large lattice ($n = 8$) experiments (Supplementary Figure 13ab).

Because our theoretical model assumes stable vortex lattice formation, it cannot be applied to relatively unstable lattices for $a = 50$ μm and $a = 90$ μm. In fact, the behavior of the persistence time τ_k for $a = 50$ μm and $a = 90$ μm clearly deviates from the exponential dependence (Supplementary Figure 13ab). Therefore, we fitted the experimental data only for $a = 60, 70, 80$ μm where stable antiferromagnetic lattices were observed (Supplementary Figure 13cd). The slopes of the exponential fitting on the semilog scale correspond to $2\eta/D$ in Eq.(12), and the ratio of η and D has weak dependence on the lattice spacing a (Supplementary Figure 13ef). Considering that the strength of noise D on the spin dynamics originates from the dynamics of the bacterial turbulence in the ROI and each spin is calculated by integrating the whole area of the corresponding ROI, we speculate that both η and D scale with the area of vortices.

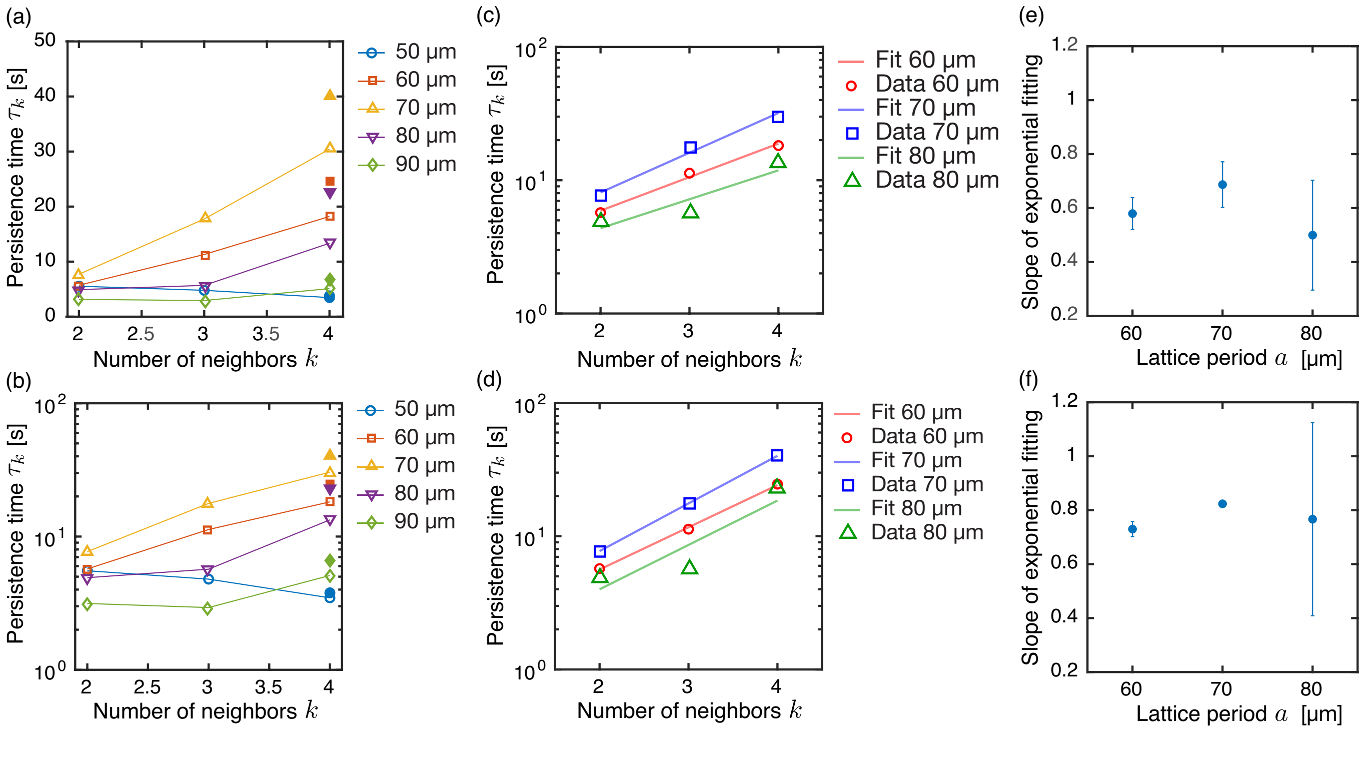

Supplementary Figure 13. (a) Persistence time τ_k vs the number of neighboring spins k for different lattice periods a. The data were obtained from the large lattice ($n = 8$) experiments shown in Fig. 3 in the main text and Supplementary Figure 4. At $k = 4$, two data points are plotted for each lattice period. The smaller ones with blank symbols were calculated from all the spins with $k = 4$ in the lattice, and the larger ones with filled symbols were calculated from the bulk spins by excluding the peripheral spins in the outer two layers of the lattices (same data as in Fig. 3c in the main text). (b) Semilog plot of the same data as in (a). (c)(d) Fitting results of the experimental data to the exponential behavior described in Eq. (12) by using (c) all the spin data and (d) the bulk spin data at $k = 4$. (e)(f) Slopes of the exponential fittings in (c) and (d) respectively as functions of lattice period a. The slopes correspond to $2\eta/D$ in Eq. (12). Slopes do not change much. Error bars: standard errors of the linear fitting on the semilog scale.

SUPPLEMENTARY REFERENCES

[1] Wioland, H., Woodhouse, F.G., Dunkel, J., and Goldstein, R.E. Ferromagnetic and antiferromagnetic order in bacterial vortex lattices, *Nat. Phys.,* **12**, 341, 2016.
