# Supplementary material for: Engineering bacterial vortex lattice via direct laser lithography
Source: Nat Commun. 2018 Oct 26;9:4486. doi: 10.1038/s41467-018-06842-6 (PMC6203773; doi:10.1038/s41467-018-06842-6)
Supplement: Supplementary file 3 — Description of Additional Supplementary Files [file 41467_2018_6842_MOESM3_ESM.pdf]

Description of Additional Supplementary Files

Supplementary Movie 1

Description: Bacterial turbulence introduced into the square small lattices of pillars. Size of pillar lattices is 4 × 4 (n = 3), while a = 50–130 µm with a 10-µm step. The movie is played at the real speed. The resolution of the movie is lowered for reducing the file size.

Supplementary Movie 2

Description: Instantaneous signs of spins overlaid in the experimental Supplementary Movie 1. Clock- wise rotations ($S_{i,a}(t) < 0$) and counterclockwise rotations ($S_{i,a}(t) > 0$) are represented by red and blue respectively as in Supplementary Figure 3. The movie is played twice slower than the real time.

Supplementary Movie 3

Description: Bacterial turbulence introduced into the square large lattices of pillars. Size of pillar lattices is 9 × 9 (n = 8), while a = 50–90 µm with a 10-µm step. The movie is played at the real time speed. The resolution of the movie is lowered for reducing the file size.

Supplementary Movie 4

Description: Instantaneous signs of spins overlaid on the experimental Supplementary Movie 3. Clock- wise rotations ($S_{i,a}(t) < 0$) and counterclockwise rotations ($S_{i,a}(t) > 0$) are represented by red and blue respectively. The movie is played at the real time speed.

Supplementary Movie 5

Description: Close up of the Supplementary Movie 4 at the lattice a = 70 µm with the original spatial resolution of the obtained experimental movie. The movie is played at twice slower than the real time speed.

Supplementary Movie 6

Description: Experiment with the hexagonal lattices. The movie is played at the real time speed. The resolution of the movie is lowered for reducing the file size. Every 2 frames of the obtained images are shown for reducing the file size.

Supplementary Movie 7

Description: Lattice size scaling experiment-part II (Supplementary Figure 10). The movie is played at the real time speed.

Supplementary Movie 8

Description: Lattice size scaling experiment –part I (Supplementary Figure 10). The movie is played at the real time speed.

Supplementary Movie 9

Description: Instantaneous signs of spins overlaid on the experimental Supplementary Movie 7. Clock- wise and counterclockwise rotations are represented by red and blue respectively. The movie is played at the real speed.

Supplementary Movie 10

Description: Instantaneous signs of spins overlaid on the experimental Supplementary Movie 8. Clockwise and counterclockwise rotations are represented by red and blue respectively. The movie is played at the real time speed.
